# Radiomics Analysis of Brain [^18^F]FDG PET/CT to Predict Alzheimer’s Disease in Patients with Amyloid PET Positivity: A Preliminary Report on the Application of SPM Cortical Segmentation, Pyradiomics and Machine-Learning Analysis

**DOI:** 10.3390/diagnostics12040933

**Published:** 2022-04-08

**Authors:** Pierpaolo Alongi, Riccardo Laudicella, Francesco Panasiti, Alessandro Stefano, Albert Comelli, Paolo Giaccone, Annachiara Arnone, Fabio Minutoli, Natale Quartuccio, Chiara Cupidi, Gaspare Arnone, Tommaso Piccoli, Luigi Maria Edoardo Grimaldi, Sergio Baldari, Giorgio Russo

**Affiliations:** 1 Nuclear Medicine Unit, ARNAS Ospedali Civico, Di Cristina e Benfratelli, 90133 Palermo, Italy; natale.quartuccio84@gmail.com (N.Q.); gasarno@interfree.it (G.A.); 2 Nuclear Medicine Unit, Fondazione Istituto G. Giglio, Contrada Pietrapollastra Pisciotto, 90015 Cefalù, Italy; riclaudi@hotmail.it; 3 Department of Nuclear Medicine, University Hospital Zurich, 8091 Zurich, Switzerland; 4 Nuclear Medicine Unit, Department of Biomedical and Dental Sciences and Morpho-Functional Imaging Nuclear Medicine Unit, University of Messina, 98122 Messina, Italy; francesco.panasiti90@gmail.com (F.P.); fabio.minutoli@unime.it (F.M.); sbaldari@unime.it (S.B.); 5 Ri.Med Foundation, Via Bandiera 11, 90133 Palermo, Italy; acomelli@fondazionerimed.com (A.C.); paolo.giaccone26@gmail.com (P.G.); 6 Institute of Molecular Bioimaging and Physiology, National Research Council (IBFM-CNR), 90015 Cefalù, Italy; alessandro.stefano@ibfm.cnr.it (A.S.); giorgio.russo@ibfm.cnr.it (G.R.); 7 Unit of Computer Systems and Bioinformatics, Department of Engineering, Università Campus Bio-Medico di Roma, Via Alvaro del Portillo 21, 00128 Rome, Italy; 8 Nuclear Medicine Unit, Department of Experimental and Clinical Biomedical Sciences “Mario Serio”, University of Florence, 50134 Florence, Italy; annachiara.arnone93@gmail.com; 9 Neurology Unit, Fondazione Istituto G. Giglio, 90015 Cefalù, Italy; chiaracupidi@gmail.com (C.C.); luigi.grimaldi@hsrgiglio.it (L.M.E.G.); 10 Unit of Neurology, Department of Biomedicine, Neurosciences and Advanced Diagnostics, University of Palermo, 90127 Palermo, Italy; tommaso.piccoli@gmail.com

**Keywords:** radiomics, Alzheimer’s disease, PET/CT, machine learning

## Abstract

Background: Early in-vivo diagnosis of Alzheimer’s disease (AD) is crucial for accurate management of patients, in particular, to select subjects with mild cognitive impairment (MCI) that may evolve into AD, and to define other types of MCI non-AD patients. The application of artificial intelligence to functional brain [^18^F]fluorodeoxyglucose (FDG) positron emission tomography (PET)/computed tomography(CT) aiming to increase diagnostic accuracy in the diagnosis of AD is still undetermined. In this field, we propose a radiomics analysis on advanced imaging segmentation method Statistical Parametric Mapping (SPM)-based completed with a Machine-Learning (ML) application to predict the diagnosis of AD, also by comparing the results with following Amyloid-PET and final clinical diagnosis. Methods: From July 2016 to September 2017, 43 patients underwent PET/CT scans with FDG and Florbetaben brain PET/CT and at least 24 months of clinical/instrumental follow-up. Patients were retrospectively evaluated by a multidisciplinary team (MDT = Neurologist, Psychologist, Radiologist, Nuclear Medicine Physician, Laboratory Clinic) at the G. Giglio Institute in Cefalù, Italy. Starting from the cerebral segmentations applied by SPM on the main cortical macro-areas of each patient, Pyradiomics was used for the feature extraction process; subsequently, an innovative descriptive-inferential mixed sequential approach and a machine learning algorithm (i.e., discriminant analysis) were used to obtain the best diagnostic performance in prediction of amyloid deposition and the final diagnosis of AD. Results: A total of 11 radiomics features significantly predictive of cortical beta-amyloid deposition (*n* = 6) and AD (*n* = 5) were found. Among them, two higher-order features (original_glcm_Idmn and original_glcm_Id), extracted from the limbic enthorinal cortical area (ROI-1) in the FDG-PET/CT images, predicted the positivity of Amyloid-PET/CT scans with maximum values of sensitivity (SS), specificity (SP), precision (PR) and accuracy (AC) of 84.92%, 75.13%, 73.75%, and 79.56%, respectively. Conversely, for the prediction of the clinical-instrumental final diagnosis of AD, the best performance was obtained by two higher-order features (original_glcm_MCC and original_glcm_Maximum Probability) extracted from ROI-2 (frontal cortex) with a SS, SP, PR and AC of 75.16%, 80.50%, 77.68%, and 78.05%, respectively, and by one higher-order feature (original_glcm_Idmn) extracted from ROI-3 (medial Temporal cortex; SS = 80.88%, SP = 76.85%, PR = 75.63%, AC = 78.76%. Conclusions: The results obtained in this preliminary study support advanced segmentation of cortical areas typically involved in early AD on FDG PET/CT brain images, and radiomics analysis for the identification of specific high-order features to predict Amyloid deposition and final diagnosis of AD.

## 1. Introduction

Alzheimer’s disease (AD) is the most common form of progressive and irreversible dementia. Early in-vivo diagnosis of AD is crucial for accurate management of patients, in particular, to select subjects with mild cognitive impairment (MCI) that may evolve into AD, and to identify MCI with suspected non-AD pathology [1]. Brain [^18^F]fluorodeoxyglucose (FDG) positron emission tomography (PET)/computed tomography(CT) is a functional neuroimaging tool evaluating dysfunction, synaptic disconnection, and neuronal loss in AD. Concerning the low membrane permeability, [^18^F]FDG dephosphorylation in the brain occurs slowly due to the low concentration of phosphatase in this tissue. Once the glucose analogue has entered the cell, it is phosphorylated in position 6 by a hexokinase; the presence of fluorine in the molecule also makes it impossible to continue along the glycolytic pathway. Therefore, brain [^18^F]FDG PET/CT has the unique ability to estimate the local cerebral metabolic rate of glucose consumption, thus providing information on the distribution of neuronal damage in AD in-vivo. Amyloid-PET/CT with several radiotracers ([^18^F]Florbetaben, [^18^F]Florbetapir, [^18^F]Flutemetamol, [^11^C]Pittsburgh compound C-PIB) provide a quantitative measure of the insoluble cortical amyloid load in vivo and it is currently being recognized to have a determining role in the diagnosis of AD. Although the target of these tracers is fibrillar Aβ, they do not represent a specific marker for a particular pool of Aβ but rather for the global cerebral amyloid load. Amyloid PET shows detectable cortical uptake with high sensitivity and specificity when a moderate-to-severe burden of plaque is present, reflecting a high negative predictive value, despite a sub-optimal specificity for possible Aβdeposition in some non-AD conditions [2]. One of the main issues regards the lack of a quantitative threshold value for the amyloid burden able to discriminate accurately patients with AD. The difficulties in identifying a precise cut-off, combined with the considerable inter-individual variability of the percentage of amyloid deposition in the population, reduce its specificity. Further, in elderly subjects with no signs of neuronal dysfunction, for example, the presence of Amyloid-PET positivity may be highlighted [3]. It must also be considered that there are other possible conditions such as Parkinson’s disease, Lewy body dementia, cerebral amyloid angiopathy, head trauma, and Down Syndrome which may show an increasein beta-Amyloid deposition [4,5,6].

The importance of exploring associated biomarkers for the early diagnosis and prediction of the disease progress of AD is a major clinical issue. The National Institute on Aging- Alzheimer’s Association (NIA-AA) proposed A/T/N diagnostic criteria in 2018, including Aβ42, p-tau, and t-tau in cerebrospinal fluid (CSF), and PET [7].However, the invasiveness of lumbar puncture for CSF assessment and the limited availability of PET with new radiotracers (for Tau and Aβ brain burden), represent a valid reason to develop new approaches with artificial intelligence applied to the more easily available methods such as FDG-PET.

The application of artificial intelligence through the development of radiomics predictive models on functional FDG-PET imaging, aiming to increase diagnostic accuracy in the diagnosis of AD is still undetermined. In this setting, we propose a radiomics analysis based on Statistical Parametric Mapping (SPM) and Pyradiomics, in combination with a Machine-Learning (ML) application, to predict Amyloid-PET positivity and diagnosis of AD. 

## 2. Materials and Methods

From July 2016 to September 2017, 43 patients (median age 64.8 years, Range 53–83 years; females = 23; males = 20; median Mini-Mental State Examination, MMSE = 19.27, Range 4–28) underwent PET/CT scans with FDG and [^18^F]Florbetaben (FBB) brain PET/CT, and at least 24 months of clinical/instrumental follow-up. Patients were retrospectively evaluated by a multidisciplinary team (MDT = neurologist, psychologist, radiologist, nuclear medicine physician, and laboratory clinic doctor) at the G. Giglio Institute in Cefalù, Italy. 

The inclusion criteria were as follows: (a) neurological and neuropsychological suspicion of neurodegenerative disease, based on the National Institute on Aging and the Alzheimer’s Association (NIA-AA) and European Federation of Neurological Societies/European Neurological Society (ENS-EFNS) criteria [8]; (b) MRI brain imaging also to rule out moderate or severe cerebrovascular defects; (c) FDG PET/CT performed for metabolic assessment; (d) availability of an amyloid-PET scan with FBB within 6 months from conventional imaging and FDG PET/CT scan; (e) report of the positivity/negativity of FBB-PET and multidisciplinary team meeting with the final diagnosis for each patient; (f) minimum duration of neurological and neuropsychological follow-up of 24 months after the first neurological evaluation for the cognitive defect.

Follow-up information were used to estimate the disease status to allow the assessment of disease progression over time and confirm/exclude the in-vivo diagnosis of AD.

### 2.1. PET/CT Acquisition Protocol

FDG PET/CT: All the subjects underwent an FDG PET/CT imaging examination using 3D PET scans, on a GE multi-ring Discovery STE PET/CT tomograph (General Electric, Milwaukee, WI, USA), at the Nuclear Medicine Unit of the G. Giglio Institute in Cefalù, Italy. All patients underwent an FDG PET before the Amyloid-PET scan. Patients received an intravenous injection of FDG (3.7 MBq/kg) at rest, in a supine position, in a quiet, dimly lit room. Image acquisition began approximately 45 min after injection, with a scan time duration of 15 min. Before the injection of FDG, the subjects were fasted for at least 6 h, and a blood glucose <160 mg/dL was measured and required to proceed with the scan. The reconstruction of the images was based on an OSEM algorithm. The low-dose CT was co-registered and used for attenuation correction.

### 2.2. Qualitative Evaluation of FDG PET

FDG PET/ CT brain transverse, sagittal and coronal images were assessed separately by two nuclear medicine physicians with expertise in PET neuroimaging. The images were classified as normal, possible, or probably suspected of AD. The rainbow scale was used to normalize the images with a uniform uptake threshold, using the basal ganglia and the cerebellum as a reference to background regions. Patients, whose cortical areas showed reduced glucose metabolism in the regions including the posterior cingulate, the precuneus, the parietal cortical territories, and the medial and lateral temporal cortex, were classified as suffering from possible or probable AD, depending on the intensity and extent of the uptake. The collection of anamnestic data played an essential role in the differential diagnosis of patients with ambiguous patterns such as AD—vascular dementia (VD), frontotemporal dementia (FTD), Lewy-body disease (DLB).

### 2.3. Image Pre-Processing and ROI Selection

The entire dataset was spatially pre-processed using the Statistical Parametric Mapping (SPM) 12 software package (https://www.fil.ion.ucl.ac.uk/spm/ (accessed on 31 January 2022)). First, each PET scan, comprising 47 Digital Imaging and Communications in Medicine (DICOM) images, was converted into a single NIfTI file, preserving the original spatial resolution. Then, the resulting 3D volume was spatially normalized to the Montreal Neurological Institute (MNI) 152 space, using the SPM unified segmentation normalization algorithm [9], which combines segmentation, bias correction and spatial normalization in a single process of optimization. This iterative method, which provides better results than simple serial applications of each step [9], allowed to directly estimate the warping tensors that register the SPM standard spatial priors (i.e., tissue probability maps) in each individual subject space. The intensity distribution of each class of tissue has been modelled by at least a mixture of two Gaussians, in order to take into account the partial volume effect; moreover, a smoothness level of 5 mm was set, in order to derive a fudge factor related to the spatial correlation between neighbouring voxels, due to the assumption of independence of the unified model [9]. The default settings were used for all other parameters. Then, the estimated nonlinear spatial transformations were applied and the PET images were resampled in a bounding box with an isotropic voxel size of 2 mm, reflecting the MNI-152 spatial proportions in a similar way to previous works [10,11,12].

After spatial normalization, we focused on four different regions of interest (ROI) that were extracted from the brain fragmentation available in SPM, whose maximum probability tissue labels derived from the “MICCAI 2012 Grand Challenge and Workshop on Multi-Atlas Labelling” (https://my.vanderbilt.edu/masi/workshops (accessed on 31 January 2022)). This neuro-anatomical classification was generated and made public by Neuromorphometrics, Inc. under academic subscription and provides a fine subdivision of cortical and non-cortical structures, for a total of 138 labels throughout the brain. Each selected ROI included 8 to 12 brain areas labelled according to Table 1 and, prior to mask extraction, their bounding box and voxel sizes were adapted to the template for alignment reasons.

### 2.4. Extraction of Radiomics Features and Machine Learning Classification

The ROIs described in Table 1 were used to extract the features through a certified and image biomarker standardisation initiative (IBSI) [12] compliant software, namely Pyradiomics [13]. Subsequently, an innovative descriptive-inferential mixed sequential approach for feature reduction and selection was used to identify a small set of radiomics features with a strong association with patient outcomes in order to obtain good predictive performance, leading to the exclusion of non-reproducible, redundant and irrelevant features from the initial set [14]. After this selection and reduction process, discriminant analysis (DA) was used as the predictive model [15,16]. The training phase was performed only once and, after being completed, the DA was able to classify new cases. Using the k-fold cross-validation strategy, the data was split into training and validation sets using a random partition. Specifically, the data were divided into k-folds: one of the folds was used as a validation set and the remaining folds were combined into the training set. The pooling was done so that both the training and validation sets maintained the same positive/negative percentage for beta-amyloid deposition compared to the original dataset. In our study, k = 5 was determined empirically by the trial-and-error method (range k: 5–15, a step of 5).

**Figure 1:**
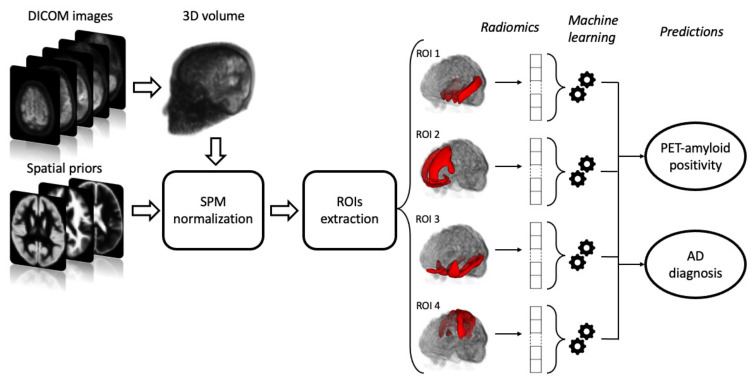
The proposed radiomics workflow, from SPM-based image pre-processing to Pyradiomics -based feature extraction process to machine learning-based classification.

Based on the above systems (Figure 1), we defined the features capable of obtaining the best diagnostic performance in predicting amyloid deposition and the final diagnosis of AD. 

## 3. Results

Forty-three patients met the inclusion criteria (Table 2). MMSE was less than 25 in 29/43 patients (Median MMSE = 19.27, Range 4–28). The clinical dementia rating (CDR) scale was greater than 0.5 in 27/43 patients. The qualitative evaluation of FDG was considered positive in 28 patients (65%), while PET with FBB was positive in 23 patients (53%). CSF assay values for quantification by Double-sandwichenzyme-linked immunosorbent assay (ELISA) were available in 18 patients, with 5 subjects showing reduced values of beta-amyloid protein (Aβ1-42) according to the cut-off < 450 pg/mL (InnotesthTAUantigen and amyloid Aβ1-42, Innogenetics). 13/18 patients, instead, showed reduced amyloid beta values (Aβ1-42) for the cutoff < 750 pg/mL; 12/18 had high CSF values of tau for the cutoff < 500 ng/L and in particular 15/18 patients phospho-tau was elevated for the cutoff < 61 pg/mL. Following the MDT evaluation, based on the results of the neuropsychological tests, the dosage of the levels of specific proteins (amyloid and tau) in the cerebrospinal fluid (CSF) if available, integration/comparison with morphological imaging (Magnetic Resonance Imaging), and evaluation of the evolution of the disease until last neurological evaluation (>24 months), 22/23 amyloid-PET positive patients were definitively classified as AD patients, while the remainder as non-AD. Similarly, 22/28 FDG PET-positive patients were classified by the multidisciplinary team as AD. 

### Analysis of Radiomics Features

Based on the SPM-based segmentation process described in the “Image pre-processing and ROI selection” section, 43 brain areas were selected for radiomics feature extraction and machine-learning classification. After the reduction and selection process based on the descriptive-inferential mixed sequential approach proposed in [14], we focused on the radiomics features capable of obtaining the best performances, expressed as sensitivity (SS), specificity (SP), precision (PR) and accuracy (AC) in the diagnostic prediction of AD in relation to the results obtained with amyloid-PET and with clinical diagnosis.

**Figure 2:**
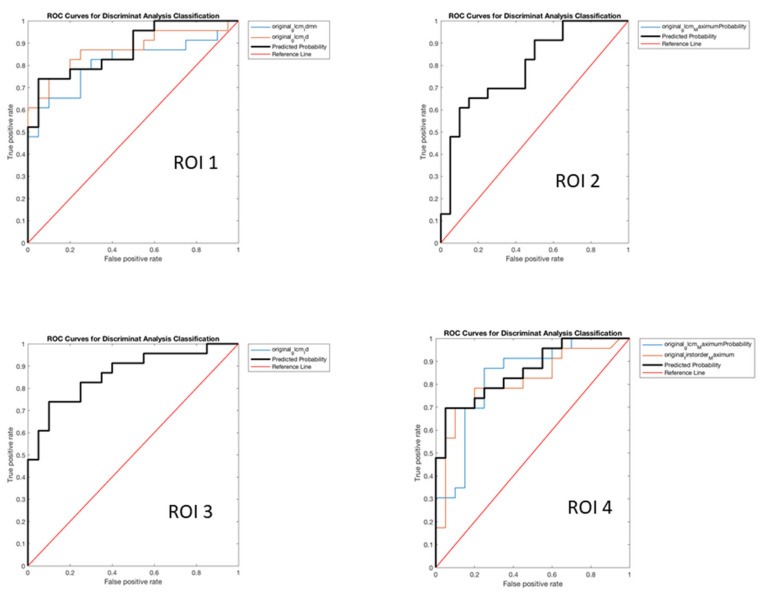
AUROC Curves of FDG-PET derived features in the prediction of Amyloid-PET positivity.

As regards the performances of prediction of PET-amyloid positivity (see Table 3), we obtained the following features, respectively, for each selected ROI (Table 3 and Figure 2):

− ROI 1
  - original_glcm_Idmn
  - original_glcm_Id: with the following values of SS 84.92%, SP 75.13%, PR 73.75%, AC 79.56% (*p* < 0.001).
− ROI 2
  - original_glcm_MaximumProbability: with the following values of SS 88.67%, SP 46.81%, PR 59.47%, AC 65.57% (*p* < 0.001).
− ROI 3
  - original_glcm_Id: with the following values of SS 93.83%, SP 61.80%, PR 67.51%, AC 76.15% (*p* < 0.001).
− ROI 4
  - original_glcm_MaximumProbability
  - original_firstorder_Maximum: with the following values of SS 86.33%, SP 64.93%, PR 66.88%, AC 74.58% (*p* < 0.001).

**Figure 3:**
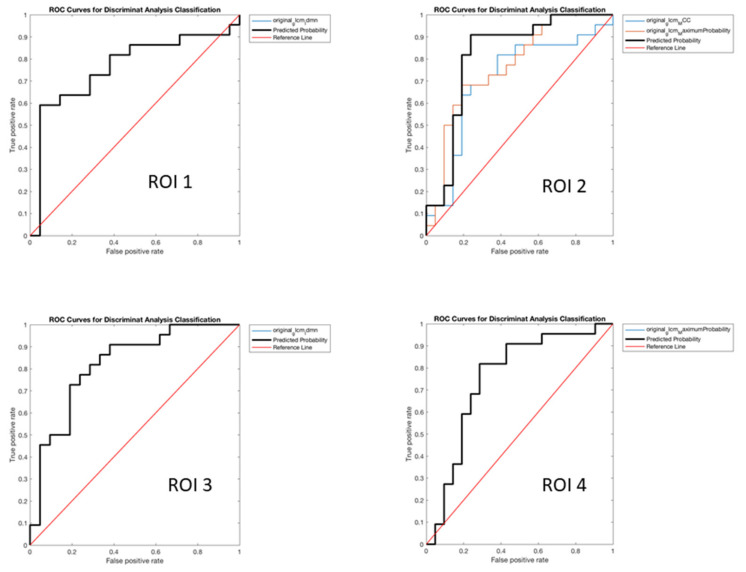
AUROC Curves of FDG-PET derived features in the prediction of AD.

As regards the performance in the prediction of the final clinical-instrumental diagnosis of AD defined by MDT evaluating all the available data, we obtained the following features from the 4 different ROIs (Table 4 and Figure 3):

− ROI 1
  - original_glcm_Idmn: with the following values of SS 66.39%, SP 57.51%, PR 58.46%, AC 61.51% and (*p* = 0.004).
− ROI 2
  - original_glcm_MCC
  - original_glcm_MaximumProbability: with the following values of SS 75.16%, SP 80.50%, PR 77.68%, AC 78.05% and (*p* = 0.002).
− ROI 3
  - original_glcm_Idmn: with the following values of SS 80.88%, SP 76.85%, PR 75.63%, AC 78.76% and (*p* < 0.001).
− ROI 4
  - original_glcm_MaximumProbability: with the following values of SS 75.50%, SP 55.25%, PR 59.53%, AC 64.96% (*p* = 0.0040).

## 4. Discussion

So far, few studies have investigated the use of artificial intelligence on FDG PET/CT brain images in the evaluation of neurodegenerative diseases. Early in-vivo diagnosis of AD is critical for accurate patient management, particularly for the selection of subjects with MCI who may evolve into AD and for defining prodromal forms of AD from other non-AD forms. Brain FDG PET/CT is a functional neuroimaging technique able to provide information on neuronal damage as dysfunction, synaptic disconnection, and neuronal loss. Amyloid-PET is currently recognized as a determinant in the diagnosis of AD in consideration of its high negative predictive value, albeit with a suboptimal specificity, determined by the possible cortical deposition of beta-amyloid in some non-AD conditions [3]. In this context, the first objectives of our study concerned the application of artificial intelligence on FDG PET/CT brain images in predicting PET-Amyloid positivity, eventually avoiding the additional execution of an amyloid-PET as a diagnostic method not widely diffuse compared to FDG-PET/CT, ultimately reducing the social and economic impact on the health system. Secondly, the opportunity to select those patients who can benefit from the diagnostic use of amyloid-PET and to evaluate how the integration of the functional and pathophysiological information of the two investigations can improve the diagnostic accuracy for AD through the help of artificial intelligence.

In the present study, through a radiomics system based on SPM, Pyradiomics, and DA to performimage normalization, the selection of the cerebral cortical areas typically involved in AD, the feature extraction process, and the development of predictive models, a total of 11 radiomics features were identified for the study objectives (6 FDG-PET features in the prediction of amyloid deposition and 5 FDG-PET features for the final diagnosis of AD). The brain FDG PET/CT metabolism alteration in the medial temporal cortex resulted in two higher-order features (original_glcm_Idmn and original_glcm_Id), as the best predictive of PET-amyloid positivity with SS, SP, PR, and AC of 84.92%, 75.13%, 73.75%, and 79.56%, respectively. The regions from which these features were extracted belonged to ROI 1, including the hippocampal, para-hippocampal, entorhinal cortex, and the territories of the middle temporal gyrus. These areas are considered in the literature as the most involved sites of AD-related functional damage in the initial/prodromal forms [17,18,19,20]. Hypometabolism in this area, therefore, appears to be the possible result of a particular correlation between functional damage in the limbic regions and a particular tropism for the cortical deposition of the beta-amyloid protein. Regarding the type of features obtained, the Gray Level Co-occurrence Matrix (GLCM) texture features belong to that group of higher-order features that have shown a significant clinical impact both in radiology and in nuclear medicine [1,21,22].

The achievement of predicting the deposition of beta-amyloid using radiomics features extracted from FDG PET/CT has not been previously reported in the literature. Furthermore, the application of artificial intelligence through the development of predictive radiomics models on functional FDG PET/CT brain imaging aimed at increasing diagnostic accuracy in the final diagnosis of AD is still highly debated. In our analysis for the prediction of AD, the best values were obtained for SS, SP, PR, and AC (75.16%, 80.50%, 77.68%, and 78.05%, respectively) for two higher-order features (original_glcm_MCC and original_glcm_MaximumProbability). The regions from which these features were extracted concerned the ROI 2, including the anterior and medial orbital gyri of the inferior frontal cortex and the medial frontal cortex. The population studied was characterized by a median age of 64.8 years and a median MMSE value of 19.27, thus configuring a good number of patients with MCI and early onset. The optimal performance of our radiomics models obtained for the limbic areas (ROI 1) in the prediction of amyloid burden and for the frontal cortex (ROI 2) in the predictive analysis for the final diagnosis of AD, could support, as noted in the scientific literature, the possible metabolic involvement of the frontotemporal synaptic connections in early-onset Alzheimer Disease (EOAD) forms. These results should be considered relevant considering the purely prognostic and non-diagnostic objective of the study.

In the same field of our study, Zhou et al. in 2019 [23], investigated the risk factors most associated with the conversion from MCI to AD, through a dual-model radiomics analysis with Cox proportional hazards, based on T1-MRI and [^18^F]FDG PET/CT scans data from the AD Neuroimaging Initiative (ADNI) database including 131 MCI patients who converted to AD and 132 MCI patients without conversion within 3 years [23]. Differently to Zhou et al., our study presents an added value in evaluating patients enrolled in the same Institute, despite the absence of the aid of linear regression analysis. However, our patients’ cohort performed the same clinical questionnaire and the same multidisciplinary evaluation, allowing a reduction of potential bias in the definition of the reference standard of positivity that may affect the innovative machine-learning tools and DA adopted in this study. We implemented the analysis using DA as a predictive model with a k-fold cross-validation strategy, first to test the prediction of FDG-PET radiomics features for PET-Amyloid positivity, and then, assessing the follow-up data of all patients, to confirm the predictive accuracy of FDG PET/CT radiomics features in defining the typical regions/patterns of hypometabolism for the diagnosis of AD. Further studies have evaluated, albeit differently, the application of radiomics models in increasing diagnostic accuracy for the diagnosis of AD, particularly in the prodromal stages of the disease, as in the article published in 2019 by Yupeng Li et al. [24]. In their multicenter study, FDG PET/CT brain data and clinical evaluations were collected in a cohort of 466 individuals (including 152 AD, 130 MCI and 184 healthy controls—HC) from the ADNI. A Support Vector Machine (SVM) was used to test the radiomics features’ ability to classify patients with HC, MCI, and AD. Brain regions were identified by ROIs distributed in the temporal, occipital, and frontal areas. A total of 168 radiomics features of AD were defined (alpha> 0.8). The classification experiment resulted in maximum accuracies of 91.5%, 83.1%, and 85.9% for the classification of AD versus HC, MCI versus HC and AD versus MCI [24]. The most evident limitation of the study, as in the previous one, was to use a predefined patient database (ADNI), with the limits resulting from a poor homogenization between different centers that inevitably use different tomographs, administration and acquisition times, in addition to the lack of clinical data from the same center for a complete clinical-instrumental evaluation. 

Another study published in October 2021 by Ping Zhou et al., proposed the application of a new deep-learning radiomics (DLR) model, based on images obtained by FDG-PET integrated with clinical parameters (DLR + C) to improve diagnostic performance and predict, according to the authors, the conversion of MCI to AD patients [25]. FDG PET/CT brain data were collected, also from the ADNI database, for a total of 168 patients with MCI who converted to AD within 3 years and 187 MCI patients without conversion within 3 years. In comparative experiments, the DLR + C method was compared with four other methods: the standard absorption value ratio (SUVR) method, the Radiomics-ROI method, the clinical method, and the clinical SUVR + method. The results obtained showed that the DLR + C model produced the best performance in terms of recognition capacity of the MCI to AD conversion with AC, SS, and SP values of 90.62 ± 1.16, 87.50 ± 0.00, and 93.39 ± 2.19%, respectively [25].

Finally, even more recently with an FDG PET study just published in January 2022, Jiang et al. evaluated MCI-to-AD conversion prediction in a dataset of 884 subjects through a radiomics- based predictive modelling (RPM) Cox model that demonstrated a better performance than that of other Cox models [26].

In comparison with our preliminary study, none of the mentioned studies implemented the analysis by comparing the results of the brain FDG PET/ CT scans in prediction of amyloid-PET positivity and then integrating these data to create a reference standardfor predicting the diagnosis of AD.

Brain FDG-PET is an established diagnostic tool for the evaluation of AD by defining the functional damage and differentiate most of the neurodegenerative disease with a crucial role in the management of patients. The implementation of AI applicated to brain FDG-PET demonstrated in this study to potentially predict Amyloid-PET positivity and AD condition with a possible future role of these methods from a prognostic tool to an augmented-diagnostic approach for the improvement of the early diagnosis.

Nevertheless, the preliminary results in our study have several limitations. First of all, the population described is unfortunately slightly inhomogeneous (MCI and prodromal AD). To obtain the results presented in a single patient a long time is still required due to the use of different software and statistical analysis, needing the involvement of informatic engineers which makes the application currently not easily reproducible in clinical practice. Furthermore, the study was limited to the comparison of FDG PET/CT with amyloid-PET and the final diagnosis, as an overall judgment of a multidisciplinary report, without a direct comparison with MRI findings, precise correlation/comparison with single clinical variables such as scores, functional tests of cognitive performance or laboratory data deriving from the analysis of CSF proteins.

## 5. Conclusions

The results obtained in this study, albeit still preliminary, support the potential experimental development of this new automated learning approach based on the extraction and selection of higher-order radiomics “features” obtained from FDG PET/CT brain images for predicting the presence of beta-amyloid deposition and the final diagnosis of AD. The preliminary results of the present study support the potential role of specific radiomics features from FDG-PET images able to improve the prognostic stratification of patients who could obtain real diagnostic benefits from amyloid-PET. Furthermore, our data suggest that specific radiomics features may improve the diagnostic AC of PET/CT in the early diagnosis of AD. We aim to increase our study cohort and the number of clinical-instrumental variables to improve the predictive models. Also, further developments in this area could concern the stratification of patients with AD based on individual sensitivity to new monoclonal therapies that are currently being validated and that could modify clinical management.

## Figures and Tables

**Table 1:** Regions of interest (ROI) extracted from the cerebral segmentation using SPM.

ROI 1	Areas	Label Index	ROI 2	Areas	Label Index	ROI 3	Areas	Label Index	ROI 4	Areas	Label Index
	Right Hippocampus	47		Right (AOrG anterior orbital gyrus	104		Right FuG fusiform gyrus	122		Right PO parietal operculum	174
	Right PHG parahippocampal gyrus	170		Right MOrG medial orbital gyrus	146		Right GRe gyrus rectus	124		Right PoG postcentral gyrus	176
	Right Ent entorhinal area	116		Right OpIFGopercular part of the inferior frontal gyrus	162		Right ITG inferior temporal gyrus	132		Right SPL superior parietal lobule	198
	Right MTG middle temporal gyrus	154		Right OrIFG orbital part of the inferior frontal gyrus	164		Right TMP temporal pole	202		Right PCgG posterior cingulate gyrus	166
	Left Hippocampus	48		Right MFC medial frontal cortex	140		Left FuG fusiform gyrus	123		Right PCuprecuneus	168
	Left PHG parahippocampal gyrus	171		Right MFG middle frontal gyrus	142		Left GRe gyrus rectus	125		Left PoG postcentral gyrus	177
	Left Ent entorhinal area	117		Left MOrG medial orbital gyrus	147		Left ITG inferior temporal gyrus	133		Left PO parietal operculum	175
	Left MTG middle temporal gyrus	155		Left AOrG anterior orbital gyrus	105		Left TMP temporal pole	203		Left SPL superior parietal lobule	199
				Left OpIFGopercular part of the inferior frontal gyrus	163					Left PCuprecuneus	169
				Left OrIFG orbital part of the inferior frontal gyrus	165					Left PCgG posterior cingulate gyrus	167
				Left MFC medial frontal cortex	141						
				Left MFG middle frontal gyrus	143						

**Table 2:** Patients’ main characteristics.

pt N°	Sex	Age	Schooling	MMSE	CDR	MRI	FDG PET	Amy-PET	Final Diagnosis (MDT)
1	F	64	21	19	1	1	1	1	1
2	M	81	5	27	0	0	0	0	0
3	F	59	8	23	0.5	1	0	0	0
4	M	63	18	21	1	1	1	1	1
5	F	79	5	20	0.5	1	0	0	0
6	F	80	5	18	2	1	1	1	1
7	F	75	5	22	1	1	1	1	1
8	F	72	5	12	1	1	1	1	1
9	F	77	5	19	2	1	0	0	0
10	F	71	13	20	2	1	1	1	1
11	F	75	5	17	2	1	1	0	0
12	F	83	5	20	1	1	0	0	0
13	M	58	18	9	2	1	1	1	1
14	F	61	13	22	2	0	0	1	1
15	M	66	13	21	1	0	1	1	1
16	F	75	8	26	0.5	1	0	0	0
17	F	53	13	13	1	1	1	1	1
18	M	66	8	28	0.5	1	1	1	1
19	M	72	18	24	0.5	1	0	0	0
20	M	79	13	17	1	1	1	1	1
21	M	69	13	28	0.5	1	1	0	0
22	F	73	13	25	1	1	1	1	1
23	M	76	8	28	0.5	1	1	0	0
24	M	74	5	29	0.5	1	0	0	0
25	M	61	18	22	2	0	1	1	1
26	F	70	8	25	1	1	1	0	0
27	F	68	13	15	2	1	1	1	1
28	M	65	8	25	0,5	1	1	1	1
29	M	80	8	18	1	1	0	0	0
30	F	71	5	4	3	0	1	1	1
31	M	78	8	13	1	1	1	0	0
32	F	74	8	10	2	1	1	1	1
33	M	80	0	18	1	1	0	0	0
34	M	78	5	22	0.5	1	0	0	0
35	M	71	0	17	1	1	1	0	0
36	M	58	8	21	1	1	1	1	1
37	F	63	18	24	1	1	0	0	0
38	F	74	5	28	0.5	1	1	1	1
39	M	77	5	30	0.5	1	0	0	0
40	M	65	8	20	1	0	1	1	1
41	M	62	17	21,46	0.5	1	1	1	0
42	F	77	5	22	1	1	1	1	1
43	F	66	8	26	0.5	1	0	0	0

Legend: N° = number; MMSE = Mini Mental State Examination; CDR = Clinical dementia rating; MRI = Magnetic Resonance Imaging.

**Table 3:** Performances of FDG-PET derived features in the prediction of Amyloid-PET positivity.

Features Selected for Each ROI	Sensitivity [%]	Specificity [%]	Precision [%]	Accuracy [%]	*p*-Value
**ROI 1**					
**original_glcm_Idmn** **original_glcm_Id**	84.92	75.13	73.75	79.56	<0.05
**ROI 2**					
**original_glcm_MCC**	88.67	46.81	59.47	65.57	<0.05
**ROI 3**					
**original_glcm_Id**	93.83	61.80	67.51	76.15	<0.05
**ROI 4**					
**original_glcm_Maximum Probability**	86.33	64.93	66.88	74.58	<0.05

**Table 4:** Performances of FDG-PET derived features in the prediction of AD.

Features Selected for Each ROI	Sensitivity [%]	Specificity [%]	Precision [%]	Accuracy [%]	*p*-Value
**ROI 1**					
**original_glcm_Idmn**	66.39	57.51	58.46	61.51	0.004
**ROI 2**					
**original_glcm_MCC** **original_glcm_MaximumProbability**	75.16	80.50	77.68	78.05	0.002
**ROI 3**					
**original_glcm_Idmn**	80.88	76.85	75.63	78.76	<0.05
**ROI 4**					
**original_glcm_Maximum Probability** **original_firstorder_Maximum**	75.50	55.25	59.53	64.96	0.004
